# A density-based clustering approach for identifying overlapping protein complexes with functional preferences

**DOI:** 10.1186/s12859-015-0583-3

**Published:** 2015-05-27

**Authors:** Lun Hu, Keith CC Chan

**Affiliations:** Department of Computing, The Hong Kong Polytechnic University, Kowloon Hong Kong, China

## Abstract

**Background:**

Identifying protein complexes is an essential task for understanding the mechanisms of proteins in cells. Many computational approaches have thus been developed to identify protein complexes in protein-protein interaction (PPI) networks. Regarding the information that can be adopted by computational approaches to identify protein complexes, in addition to the graph topology of PPI network, the consideration of functional information of proteins has been becoming popular recently. Relevant approaches perform their tasks by relying on the idea that proteins in the same protein complex may be associated with similar functional information. However, we note from our previous researches that for most protein complexes their proteins are only similar in specific subsets of categories of functional information instead of the entire set. Hence, if the preference of each functional category can also be taken into account when identifying protein complexes, the accuracy will be improved.

**Results:**

To implement the idea, we first introduce a preference vector for each of proteins to quantitatively indicate the preference of each functional category when deciding the protein complex this protein belongs to. Integrating functional preferences of proteins and the graph topology of PPI network, we formulate the problem of identifying protein complexes into a constrained optimization problem, and we propose the approach DCAFP to address it. For performance evaluation, we have conducted extensive experiments with several PPI networks from the species of Saccharomyces cerevisiae and Human and also compared DCAFP with state-of-the-art approaches in the identification of protein complexes. The experimental results show that considering the integration of functional preferences and dense structures improved the performance of identifying protein complexes, as DCAFP outperformed the other approaches for most of PPI networks based on the assessments of independent measures of f-measure, Accuracy and Maximum Matching Rate. Furthermore, the function enrichment experiments indicated that DCAFP identified more protein complexes with functional significance when compared with approaches, such as PCIA, that also utilize the functional information.

**Conclusions:**

According to the promising performance of DCAFP, the integration of functional preferences and dense structures has made it possible to identify protein complexes more accurately and significantly.

## Background

Protein complexes are biomolecules that contain a number of proteins interacting with each other to perform different cellular functions [1]. Hence, protein complexes discovered in PPI networks can lead to a better understanding of the roles of proteins in different cellular systems. It is for this reason that the problem of identifying protein complexes has been being popular over the last decade. To tackle it, a considerable number of techniques, including both laboratory-based and computational techniques, have been proposed.

Laboratory-based techniques have been developed to identify protein complexes with laboratory experiments, such as chemical cross-linking [2,3], AP-MS [4,5] and two-hybrid systems [6,7]. Though promising, laboratory-based techniques are not satisfactory in terms of efficiency. Taking AP-MS as an example, identifying different protein complexes requires to perform a series of experiments using different bait protein every time [8]. Moreover, for laboratory-based techniques the set of protein complexes that can be identified by them is usually incomplete, as some protein complexes may not be able to be discovered under current experimental conditions [9].

To avoid the problems of laboratory-based techniques, a number of different computational approaches have been proposed as a complementary tool of identifying protein complexes and they are, by and large, developed based on the use of different graph clustering algorithms. In general, by representing a PPI network as a graph where vertices represent proteins and edges are the interactions between proteins, the resultant graph clusters are considered as the identified protein complexes. To do so, computational approaches that purely rested on the graph topology of PPI network discovered graph clusters by following certain topological structures, such as density [10-12], k-cores [13,14], core-attachment structures [15,16] and peripheries [17], [18]. A detailed literature review of these topology-based approaches can be referred to our previous work [19].

Recently, the functional information of proteins has become available and easy-to-access due to the development of online resources [20-22], thus providing an alternative view to identify protein complexes. For proteins in the same protein complex, it is known that they may have similar protein functional information [23], so certain efforts have been made to identify protein complexes by additionally taking such information into consideration. In particular, Lubovac et al. [24] introduced two alternative network measures that combined functional information with topological properties to weight proteins and then identified protein complexes based on proteins with high weights; Wang et al. [25] evaluated the reliability of PPIs according to the similarity of functional information and developed a cluster-expanding algorithm to detect protein complexes with core-attachment structure; Zhang et al. [26] constructed an augmented network from the original PPI network by integrating with the functional information so that protein complexes could be discovered based on cliques identified from the augmented network; Hu et al. [19] weighted the PPI network based on the dependency of functional information and then applied a Markov clustering process to identify protein complexes; Zhang et al. [27] performed the task of identifying protein complexes by proposing a generative model to combine both functional and topological information. In addition to the use of functional information, Wu et al. [28] made use of biological data from multiple resources, such as Gene Ontology (GO), gene expression profiles and AP-MS data, to assess the affinity of two proteins, and then identified protein complexes based on these affinity scores.

Obviously, the aforementioned approaches make use of the functional information of proteins by designing different measures so as to weight the PPIs or proteins from different perspectives, such as similarity [24-26], dependency [19] and probability distribution [27]. Though efficient, these approaches have a noticeably common disadvantage as they design their measures by taking all the functional categories as an integrity while ignoring the preferences of individual categories of the functional information. For functional information, the GO project [20,29] describes it in three functional categories from different perspectives of proteins and they are biological processes, molecular functions and cellular components. According to our previous researches [19,30], for most protein complexes their proteins are only similar in specific subsets of these three functional categories instead of all of them. Hence, when deciding whether two proteins should be classified into the same complex, the functional categories where proteins are similar should be considerably more preferred than those where proteins are not similar. As existing approaches that additionally consider the functional information of proteins cannot distinguish such difference, we believe that the full potential knowledge of the functional information of proteins is yet to be exploited in order to identify protein complexes accurately. In this regard, if we have a way to make it possible that proteins in the same protein complex can be identified by emphasizing the functional categories they are similar while ignoring the functional categories they are not similar, the performance of identifying protein complexes can be further improved.

To do so, we propose DCAFP that can identify protein complexes based on the following two intuitive properties:

1. Proteins in the same protein complex are densely connected from the topological perspective;
2. Proteins in the same protein complexes are at least similar in specific subsets of functional categories from the perspective of functional information.

It is seen that the first property requires that graph clusters of interest should have a dense topological structure which has been widely adopted to identify protein complexes and the second property is to emphasize the necessity of considering the individual preferences of functional categories when identifying protein complexes from the perspective of functional information.

Regarding the implementation of DCAFP, we employ a likelihood matrix to represent to what extent pairwise proteins are likely to be identified in the same protein complex. If the likelihood between two proteins is high, it means that they are more likely to be identified in the same protein complex by DCAFP. Another benefit of using the likelihood matrix is that we may be able to identify overlapping protein complexes. To indicate the preference of each functional category when forming protein complexes, we assign a corresponding preference vector to each of proteins. With this preference vector, the individual preferences of functional categories can be quantitatively indicated when identifying the protein complex the corresponding protein belongs to. Then following the intuitive properties aforementioned, we formulate a constrained optimization problem to identify protein complexes based on the likelihood matrix and the preference vectors of proteins. This optimization problem is addressed by DCAFP adopting the strategy of alternatively optimizing the likelihood matrix and preference vectors through an iterative procedure. This procedure initially starts from a random guess of both the likelihood matrix and the preference vectors of all proteins and then iteratively improves the quality of the clustering until convergence.

The performance of DCAFP has been evaluated by using five PPI networks from two species of Saccharomyces cerevisiae and Human and the three functional categories of GO. The extensive experimental results not only show that DCAFP has a promising performance when compared with state-of-the-art approaches but also demonstrate the ability of DCAFP to identify overlapping protein complexes.

## Methods

### Mathematical preliminaries

To represent a PPI network, we use a 3-element tuple G = {V, E, Λ}, where V = {*v*_*i*_} (1 ≤ *i* ≤ *n*_V_) is a set of *n*_V_ proteins, E = { *e*_*ij*_} denotes all the *n*_E_ interactions, and *Λ* = {Λ_*p*_, Λ_*f*_, Λ_*c*_} is a set of the three functional categories, i.e., biological processes*,* molecular functions and cellular components. An interaction *e*_*ij*_ ∈ E connects the two proteins *v*_*i*_ and *v*_*j*_ in G. To represent the topology of G, we use an adjacency matrix $\mathbf{T}=\left[\ {t}_{ij}\right]\in {\left\{0,\ 1\right\}}^{n_{\mathrm{V}}\times {n}_{\mathrm{V}}}$, where *t*_*ij*_ = 1 if *e*_*ij*_ ∈ E, *t*_*ij*_ = 0 otherwise. For an arbitrary functional category, say Λ_*p*_ ∈ *Λ*, we define a domain *dom* (Λ_*p*_) as a set of possible values that can be taken by Λ_*p*_. In the GO database, *dom* (Λ_*p*_) is the set of GO terms in Λ_*p*_.

We use a likelihood matrix **W** = [*w*_*ij*_] (1 ≤ *i*, *j* ≤ *n*_V_) to represent the likelihoods of being grouped in the same cluster for all pairwise proteins in G. By the definition of **W**, we have *w*_*ij*_ ∈ [0, 1] denoting how likely *v*_*i*_ and *v*_*j*_ are being identified in the same cluster. The larger the value of *w*_*ij*_ is, the more likely *v*_*i*_ and *v*_*j*_ are grouped in the same cluster.

To indicate the similarity of proteins in each functional category, a set of similarity matrices **A** = {**A**_*p*_, **A**_*f*_, **A**_*c*_} is adopted. Taking ${\mathbf{A}}_p=\left[{a}_{ij}^p\right]\ \left(1\le i,j\le {n}_{\mathrm{V}}\right)$ as an example, we use it to represent the similarity matrix of Λ_*p*_ and ${a}_{ij}^p$ denotes the similarity score of *v*_*i*_ and *v*_*j*_ in terms of Λ_*p*_.

In addition to **W** and **A**, we also have another matrix **D** = [*d*_*ij*_] (1 ≤ *i*, *j* ≤ *n*_V_) used to represent the similarity between any two proteins from the perspective of topological structure. Assuming that for *v*_*i*_ we have V_*i*_ = {*v*_*k*_|*e*_*ik*_ ∈ E} representing the set of interacting proteins of *v*_*i*_ and so is V_*j*_ for *v*_*j*_, the value of *d*_*ij*_ is the percentage of common proteins found in both V_*i*_ and V_*i*_. Obviously the more interacting proteins *v*_*i*_ and *v*_*j*_ have in common, the larger the value of *d*_*ij*_ is.

To show the individual preferences of functional categories during clustering, we use a preference vector of functional categories for each of proteins and denote it as ${\mathbf{r}}_i^{\mathrm{T}}=\left({r}_{ip},{r}_{if},{r}_{ic}\right)$ w.r.t. *v*_*i*_. In **r**_*i*_, each element is a non-negative value and we have the constraint *r*_*ip*_ + *r*_*if*_ + *r*_*ic*_ = 1. With **r**_*i*_, we are able to quantify to what extent functional categories in *Λ* are preferred when we determine whether another protein *v*_*j*_ should be grouped in the same cluster as *v*_*i*_ from the perspective of functional information. In other words, regarding the clustering related to *v*_*i*_, *Λ*_*p*_ will play a more important role if *r*_*ip*_ is assigned a larger value. To represent the preference vectors of all proteins in V, we use the preference matrix $\mathbf{R}={\left({\mathbf{r}}_1,{\mathbf{r}}_2,\dots, {\mathbf{r}}_{n_{\mathrm{v}}}\right)}^{\mathrm{T}}$.

The problem of identifying protein complexes is to identify a set of clusters C = {C_*p*_} (C_*p*_ ⊆ V) from G. In each cluster of C, proteins are densely connected and they are similar in specific subsets of *Λ*. Since DCAFP is capable of identifying overlapping protein complexes, we may have ∃ C_*p*_, C_*q*_ ∈ C : C_*p*_ ∩ C_*q*_ ≠ ∅.

### Problem formulation

Given **T**, **A** and **D**, we target to find appropriate **W** and **R** so that the resultant clusters can best satisfy the aforementioned intuitive properties. Following this idea, we formulate an optimization problem as:

1 $$ \begin{array}{l} \max J\left(\mathbf{W},\mathbf{R}\right)=\\ {}Tr\left({\left({\mathbf{W}}_{\mathbf{T}}\right)}^{\mathrm{T}}{\mathbf{W}}_{\mathbf{D}}{\mathbf{W}}_{\mathbf{T}}\right)+Tr\left({\displaystyle \sum_{m\in \left\{p,\ f,\ c\right\}}{\mathbf{W}}^{\mathrm{T}}{\mathbf{S}}_m}\right)-{\left\Vert \mathbf{W}\right\Vert}_F^2-{\left\Vert \mathbf{R}\right\Vert}_F^2\\ {}s.t.\kern0.75em \mathbf{R}\mathbf{1}=\mathbf{1},\ \mathbf{R}\ge 0,\ 0\le \mathbf{W}\le 1\end{array} $$

where **W**_**T**_ = **T** ∘ **W** is the entrywise product of **T** and **W**, **W**_**D**_ = **D** ∘ **W** is the entrywise product of **D** and **W**, Tr is the trace expression of the corresponding matrix, ${\left\Vert \mathbf{W}\right\Vert}_F^2=Tr\left({\mathbf{W}}^{\mathrm{T}}\mathbf{W}\right)$ is the squared Frobenius norm of **W**, ${\left\Vert \mathbf{R}\right\Vert}_F^2=Tr\left({\mathbf{R}}^{\mathrm{T}}\mathbf{R}\right)$ is the squared Frobenius norm of **R**, **1** is a column vector with a proper size and each element of **1** is 1, and ${\mathbf{S}}_m=\left[{s}_{ij}^m\right]$ is a *n*_V_ × *n*_V_ matrix each cell of which is defined as ${s}_{ij}^m={a}_{ij}^m\ {r}_{im}$.

The optimization problem as described by (1) consists of three components: a measure of clustering quality, regularizations and constraints. To clarify the eligibility of the optimization problem of (1) in terms of satisfying the aforementioned intuitive properties, we give a detailed analysis of (1) so that the eligibility can be proved.

To confirm the topological structures of clusters identified, we constraint our analysis on the first term of (1) and rewrite it by following the trace expression as:

2 $$ \partial Tr\left({\left({\mathbf{W}}_{\mathbf{T}}\right)}^{\mathrm{T}}{\mathbf{W}}_{\mathbf{D}}{\mathbf{W}}_{\mathbf{T}}\right)={\displaystyle \sum_{i=1}^{n_{\mathrm{V}}}{\displaystyle \sum_{j=1}^{n_{\mathrm{V}}}\Big({d}_{ij}{w}_{ij}\times {\displaystyle \sum_{k=1}^{n_{\mathrm{V}}}w{}_{ik}w_{jk}{t}_{ik}{t}_{jk}}}}\Big). $$

According to the definition of **D**, we know that a large value of *d*_*ij*_ indicates that *v*_*i*_ and *v*_*j*_ have a large percentage of proteins in common. For a third protein *v*_*k*_, *w*_*ik*_ *w*_*jk*_ denotes the degree of being grouped in the same cluster with both *v*_*i*_ and *v*_*j*_ while *t*_*ik*_ *t*_*jk*_ ensures that *v*_*k*_ contributes to the value of *Tr*((**W**_**T**_)^T^ **W**_**D**_ **W**_**T**_) only if both *e*_*ik*_ and *e*_*jk*_ are found in E. It is not difficult to conclude that if two proteins have many common proteins most of which are also likely to be grouped in the same cluster as the two proteins we concern, the value of (1) is to be maximized. Therefore, this conclusion, to some extent, assure that proteins in the same cluster are densely connected.

For the second term in (1), we use it to manipulate the functional information during clustering so that clusters can be identified based on a subset of functional categories with high preferences. To prove it, the second terms of (1) is rewritten with trace expression in terms of *r*_*im*_ and *w*_*ij*_ as below:

3 $$ Tr\left({\displaystyle \sum_{m=1}^{n_{\varLambda }}{\mathbf{W}}^{\mathrm{T}}{\mathbf{S}}_m}\right)=\beta {\displaystyle \sum_{i=1}^{n_{\mathrm{V}}}{\displaystyle \sum_{m=1}^{n_{\varLambda }}\left({r}_{im}\times {\displaystyle \sum_{j=1}^{n_{\mathrm{V}}}{a}_{ij}^m{w}_{ij}}\right)}}, $$

4 $$ Tr\left({\displaystyle \sum_{m=1}^{n_{\varLambda }}{\mathbf{W}}^{\mathrm{T}}{\mathbf{S}}_m}\right)=\beta {\displaystyle \sum_{i=1}^{n_{\mathrm{V}}}{\displaystyle \sum_{j=1}^{n_{\mathrm{V}}}\left({w}_{ij}\times {\displaystyle \sum_{m=1}^{n_{\varLambda }}{a}_{ij}^m{r}_{im}}\right)}}. $$

According to (3) and (4), we will take *v*_*i*_ ∈ V as an example to explain how *r*_*im*_ and *w*_*ij*_ are supposed to be determined in order to maximize (1). In (3), given constraints ${\displaystyle {\sum}_{m=1}^{n_{\varLambda }}{r}_{im}}=1$ and *r*_*im*_ > 0**,** the preference vector of *v*_*i*_, i.e., **r**_*i*_, should assign more weights (i.e., *r*_*im*_) to categories where large similarity scores (i.e., ${a}_{ij}^m$) between *v*_*i*_ and other proteins occur more frequently. The trace expression in (4) shows that *w*_*ij*_ ought to be with a large value if the amount of similarity scores between *v*_*i*_ and *v*_*j*_ (i.e., ${\displaystyle {\sum}_{m=1}^{n_{\varLambda }}{a}_{ij}^m{r}_{im}}$) is also large. In sum, combining the meanings of (3) and (4), the term $\beta\ Tr\left({\displaystyle {\sum}_{m=1}^{n_{\varLambda }}{\mathbf{W}}^{\mathrm{T}}{\mathbf{S}}_m}\right)$ allows us to identify clusters from a subset of functional categories that are with high preference values.

After discussing the appropriateness of the first two terms of (1) as being an eligible measure of clustering quality, the other two terms in (1) are related to the regularizations of **W** and **R** respectively. For **W**, we use ${\left\Vert \mathbf{W}\right\Vert}_F^2$ to raise the penalty for the case that the values of all items in **W** are moving toward the maximum value (i.e., 1). The term ${\left\Vert \mathbf{R}\right\Vert}_F^2$ is to regularize the smoothness of each preference vector in **R**.

### Solution

To determine **W** and **R** that can maximize (1), we adopt the strategy of optimizing **W** and **R** alternatively. That is to say, at each iteration, DCAFP first updates **R** while keeping **W** fixed and then use the updated **R** to update **W**. Assuming that we are now at the (*l* + 1)_*th*_ iteration with **W** ^(*l*)^ and **R** ^(*l*)^ available for use, the details of obtaining **W** ^(*l* + 1)^ and **R** ^(*l* + 1)^ are presented as below.

### *Updating* R

To facilitate understanding, we now use max *J*(**R**|**W**) to denote that *J*(**W**, **R**) is about to be maximized by updating **R** with a fixed **W**. First of all, we formulate a sequence of quadratic subproblems for approximating the maximization of *J*(**R**|**W**) as:

5 $$ \max J\left(\mathbf{R}\Big|\mathbf{W}\right)= \max {\displaystyle \sum_{i=1}^{n_{\mathrm{V}}}J\left({\mathbf{r}}_i\Big|\mathbf{W}\right)}={\displaystyle \sum_{i=1}^{n_{\mathrm{V}}} \max J\left({\mathbf{r}}_i\Big|\mathbf{W}\right)}. $$

In (5), each subproblem max *J*(**r**_*i*_|**W**) is designed to maximize *J*(**R**|**W**) in terms of **r**_*i*_. Therefore, the problem of updating **R** is divided into several subproblems each of which is to update the corresponding **r**_*i*_ as a part of the solution of (1).

To solve max *J*(**r**_*i*_|**W**), we employ the primal-dual active set strategy [31] that is known to be efficient to solve constrained optimal problems to search for the feasible improving directions of **r**_*i*_. In particular, given ${\mathbf{r}}_i^{(l)}$, an optimal move $\Delta {\mathbf{r}}_i^{\left(l+1\right)}$ toward ${\mathbf{r}}_i^{\left(l+1\right)}$ should be able to maximize $J\left({\mathbf{r}}_i^{(l)}+\Delta {\mathbf{r}}_i^{\left(l+1\right)}\Big|{\mathbf{W}}^{(l)}\right)$. Hence, after some algebraic manipulations based on the Karush–Kuhn–Tucker (KKT) linear conditions, $\Delta {\mathbf{r}}_i^{\left(l+1\right)}$ can be derived as

6 $$ \Delta\ {r}_{im}^{\left(l+1\right)}=\left\{\begin{array}{l}0, \kern2.25em m\in {\varGamma}_{\ge}\cap {\mathrm{P}}_i^{\left(l+1\right)}\\ {}\frac{\partial\ J\left({\mathbf{r}}_i\Big|{\mathbf{W}}^{(l)}\right)}{\partial\ {r}_{im}}-\frac{{\displaystyle {\sum}_{m\ \in\ {\overline{{\mathrm{P}}_i}}^{\left(l+1\right)}}\frac{\partial\ J\left({\mathbf{r}}_i\Big|{\mathbf{W}}^{(l)}\right)}{\partial\ {r}_{im}}\ }}{n_{\varLambda }-{n}_{{\mathrm{p}}_i}^{\left(l+1\right)}+1},\ \mathrm{otherwise}\end{array}\right. $$

where *Γ*_≥_ is the set of greater-than constraints of **r**_*i*_, ${\mathrm{P}}_i^{\left(l+1\right)}$ is the set of active constraints at (*l* + 1)_*th*_ iteration, ${n}_{{\mathrm{p}}_i}^{\left(l+1\right)}$ is the size of ${\mathrm{P}}_i^{\left(l+1\right)}$, ${\overline{{\mathrm{P}}_{\mathrm{i}}}}^{\left(l+1\right)}$ is the set of inactive constraints at (*l* + 1)_*th*_ iteration.

In (6), $\frac{\partial\ J\left({\mathbf{r}}_i\Big|{\mathbf{W}}^{(l)}\right)}{\partial\ {r}_{im}}=-\kern0.5em {r}_{im}^{(l)}+{\displaystyle {\sum}_{j=1}^{n_{\mathrm{V}}}{w}_{ij}^{(l)}{a}_{ij}^m}$. Therefore, regarding the subproblem max *J*(**r**_*i*_|**W**), we can obtain $\Delta {\mathbf{r}}_i^{\left(l+1\right)}$ with (6). If $\Delta {\mathbf{r}}_i^{\left(l+1\right)}\ne 0$, an usual update of ${\mathbf{r}}_i^{\left(l+1\right)}$ can be made with ${\mathbf{r}}_i^{\left(l+1\right)}={\mathbf{r}}_i^{(l)}+\Delta {\mathbf{r}}_i^{\left(l+1\right)}$ so that max *J*(**r**_*i*_|**W**) is optimized over the active constraints. However, a full step in the direction $\Delta {\mathbf{r}}_i^{\left(l+1\right)}$ may cause some inactive constraints to be violated as we only consider the active constraints related to **r**_*i*_. To avoid it, we have to find the maximum step ${\lambda}_i^{\left(l+1\right)}$ that we can take for the update of ${\mathbf{r}}_i^{\left(l+1\right)}$ in the direction $\Delta {\mathbf{r}}_i^{\left(l+1\right)}$. In particular, ∀ *m* ∈ *Γ*_≥_, if $\Delta {r}_{im}^{\left(l+1\right)} < 0$, the condition ${r}_{im}^{(l)}+{\lambda}_{im}^{\left(l+1\right)}\cdot \Delta {r}_{im}^{\left(l+1\right)}\ge 0$ must be satisfied so that the update on ${r}_{im}^{\left(l+1\right)}$ will not violate the constraint ${\mathbf{1}}_m^{\mathrm{T}}{\mathbf{r}}_i\ge 0$. For ${\lambda}_i^{\left(l+1\right)}$, we can determine it as:

7 $$ {\lambda}_i^{\left(l+1\right)}= \min \left\{1,\kern0.5em  \min \left\{{\lambda}_{im}^{\left(l+1\right)}=\frac{r_{im}^{(l)}}{-\Delta {r}_{im}^{\left(l+1\right)}}:m\in {\varGamma}_{\ge },\Delta {r}_{im}^{\left(l+1\right)}<0\right\}\right\} $$

where 1 accounts for the equality constraint of **r**_*i*_ as defined in (1). Then ${\mathbf{r}}_i^{\left(l+1\right)}$ can be determined according to (8).

8 $$ {\mathbf{r}}_i^{\left(l+1\right)}={\mathbf{r}}_i^{(l)}+{\lambda}_i^{\left(l+1\right)}\cdot \Delta {\mathbf{r}}_i^{\left(l+1\right)} $$

Once applying (8) to all preference vectors in **R**, **R** ^(*l* + 1)^ is obtained.

### *Updating* W

Similar to the update of **R**, we use *J*(**W**|**R**) to represent the optimization problem of *J*(**W**, **R**) in terms of **W** by fixing **R**. Observing (1), we find that each element of **W** is independent with others as there are no constraints between any two elements in **W**. Therefore, we can approximate max *J*(**W**|**R**) as:

9 $$ \begin{array}{l} \max\ J\left(\mathbf{W}\Big|\mathbf{R}\right)= \max {\displaystyle \sum_{i=1}^{n_{\mathrm{V}}}{\displaystyle \sum_{j=1}^{n_{\mathrm{V}}}J\left({w}_{ij}\Big|\mathbf{R}\right)}}={\displaystyle \sum_{i=1}^{n_{\mathrm{V}}}{\displaystyle \sum_{j=1}^{n_{\mathrm{V}}} \max J\left({w}_{ij}\Big|\mathbf{R}\right)}}.\\ {}s.t.\kern0.5em 0\le {w}_{ij}\le 1\end{array} $$

where *J*(*w*_*ij*_|**R**) is given in (10).

10 $$ \begin{array}{l}J\left({w}_{ij}\Big|\mathbf{R}\right)=-{w}_{ij}^2 + {d}_{ij}{w}_{ij}{\displaystyle \sum_{k=1}^{n_{\mathrm{V}}}{w}_{ik}{w}_{jk}{t}_{ik}{t}_{jk}}+{w}_{ij}{\displaystyle \sum_{m=1}^{n_{\varLambda }}{s}_{ij}^m}\\ {}\kern4.25em =-{w}_{ij}^2+{w}_{ij}\left({d}_{ij}{\displaystyle \sum_{k=1}^{n_{\mathrm{V}}}{w}_{ik}{w}_{jk}{t}_{ik}{t}_{jk}} + {\displaystyle \sum_{m=1}^{n_{\varLambda }}{s}_{ij}^m}\right)\end{array} $$

From (10), the problem of max *J*(**W**|**R**) is converted into a sequence of subproblems w.r.t. *w*_*ij*_. In fact, the subproblem max *J*(*w*_*ij*_|**R**) is essentially a maximization issue as indicated by (10). Because $\frac{d^2J\left({w}_{ij}\Big|\mathbf{R}\right)}{d\ {w_{ij}}^2}= - 1<0$, (10) is a concave function with respect to *w*_*ij*_. It is easy to conclude that the maximum value of (10) will be obtained when $\frac{d\ J\left({w}_{ij}\Big|\mathbf{R}\right)}{d\ {w}_{ij}}=0$ if without the constraint 0 ≤ *w*_*ij*_ ≤ 1. Assuming that ${w}_{ij}^{\ast }$ is the value of *w*_*ij*_ that satisfies the equation $\frac{d\ J\left({w}_{ij}\Big|{\mathbf{R}}^{\left(l+1\right)}\right)}{d\ {w}_{ij}}=0$, we have ${w}_{ij}^{\ast }={d}_{ij}{\displaystyle \sum_{k=1}^{n_{\mathrm{V}}}{w}_{ik}^{(l)}{w}_{jk}^{(l)}{t}_{ik}{t}_{jk}}+{\displaystyle \sum_{m=1}^{n_{\varLambda }}{a}_{ij}^m{r}_{im}^{\left(l+1\right)}}$. Therefore at (*l* + 1)_*th*_ iteration, the solution to the subproblem $\max J\left({w}_{ij}^{\left(l+1\right)}\Big|{\mathbf{R}}^{\left(l+1\right)}\right)$ when considering the constraint $0\le {w}_{ij}^{\left(l+1\right)}\le 1$ is given in (11).

11 $$ {w}_{ij}^{\left(l+1\right)} = \left\{\begin{array}{l}0,\kern3em {w}_{ij}^{\ast}\le 0\\ {}{w}_{ij}^{\ast },\kern1.75em 0<{w}_{ij}^{\ast }<1\\ {}\ 1,\kern3.25em {w}_{ij}^{\ast}\ge 1\end{array}\right. $$

So far, **R** ^(*l* + 1)^ and **W** ^(*l* + 1)^ are able to be derived from **R** ^(*l*)^ and **W** ^(*l*)^ with (8) and (11) respectively at (*l* + 1)_*th*_ iteration, we will explain the details of DCAFP in the next subsection.

### DCAFP

DCAFP has three steps: 1) finding the optimal solution of max *J*(**W**, **R**), 2) identifying base clusters, and 3) obtaining C by merging these base clusters.

**Figure 1:**
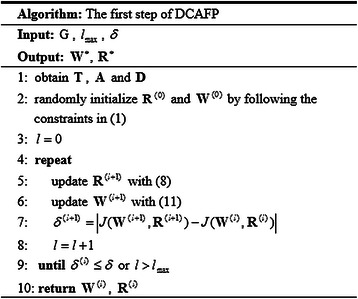
A complete description of the first step of DCAFP.

In the first step, DCAFP adopts an iteration procedure to search for a local optimum of the optimization problem of max *J*(**W**, **R**). At the (*l* + 1)_*th*_ iteration, the previous results of **R** and **W,** i.e., **R** ^(*l*)^ and **W** ^(*l*)^, will be used to reestimate **R** ^(*l* + 1)^ and **W** ^(*l* + 1)^ according to (8) and (11). The iteration procedure will be terminated if a convergence of max *J*(**W**, **R**) is reached or the procedure is now at the maximum number of iterations *l*_max_. Regarding the convergence of max *J*(**W**, **R**), the difference between *J*(**W** ^(*l* + 1)^, **R** ^(*l* + 1)^) and *J*(**W** ^(*l*)^, **R** ^(*l*)^) should not be more than a predefined threshold, i.e., *δ*. Once converged, the current matrices of **W** and **R** will be taken as the solution to max *J*(**W**, **R**) and represented as **W** ^∗^ and **R** ^∗^ respectively. A complete description of the first step of DCAFP is given in Figure 1.

Given **W** ^∗^, we now define base clusters as subgraphs where interacting proteins have a likelihood value not less than a predefined threshold, i.e., *w*_min_. That is to say, two proteins *v*_*i*_ and *v*_*j*_ are grouped in the same base cluster iff *w*_*ij*_ *t*_*ij*_ ≥ *w*_min_. To find all base clusters in G, DCAFP performs a heuristic search that is similar to [32]. In particular, DCAFP takes each of proteins in V as the seed of a base cluster and then applies a depth-first search starting from this seed so as to obtain the corresponding base cluster. After removing duplicate base clusters, the second step of DCAFP is completed.

The final step of DCAFP merges base clusters with a high degree of overlap in their proteins. For two base clusters BC_*p*_ and BC_*q*_ (BC_*p*_, BC_*q*_ ⊆ V), the overlapping score between them is defined as:

12 $$ os\left({\mathrm{BC}}_p,{\mathrm{BC}}_q\right)=\frac{{\mathrm{BC}}_p\cap {\mathrm{BC}}_q}{{\mathrm{BC}}_p\cup {\mathrm{BC}}_q}. $$

In essence, DCAFP merges the base clusters using the equivalent of a single-link clustering algorithm [33] where a predetermined maximal overlapping score, i.e., *os*_max_, between base clusters serves as the terminating criterion. This clustering algorithm is incremental and order independent. This step creates clusters that are more coherent and also reduces the fragmentation of the resultant clusters in C.

With the three steps mentioned above, DCAFP can complete the task of identifying protein complexes by obtaining C.

## Results and discussions

### Data collections

For the purpose of performance evaluation, DCAFP has been tested with five sets of real PPI network data, including Krogan PPI network [23], Gavin PPI Network [34], Collins PPI network [35], DIP Scere PPI network [36] and DIP Hsapi network [36]. In particular, Kogran, Gavin, Collins and DIP Scere PPI networks were obtained from the yeast Saccharomyces cerevisiae while DIP Hsapi network was collected from Human. Obviously the first four PPI networks overlapped with each other to different extents as they were from the same species. We chose to use more than one PPI networks of Saccharomyces cerevisiae as they were all different in terms of unreliability resulted from different PPI identification processes [37]. To assess the robustness of DCAFP against the unreliability, we therefore preferred to use four yeast PPI networks even though there were some overlaps among them.

The data of PPI networks of Krogan, Gavin and Collins was provided by the BioGRID database [38] with version 3.2.118. For DIP Scere and DIP Hsapi networks, we obtained them from the snapshot of the DIP database [36] as of April 6, 2013, which was the latest version when we were working on this paper. We removed all self-connecting interactions and duplicated interactions as a data pre-processing of these PPI networks. The details of all these five PPI networks are presented in Table 1.

**Table 1:** **The details of PPI networks used in experiments**

	***n***_**V**_	***n***_**E**_	**Density** ^*^
Kogran	2674	7075	0.002
Gavin	1430	6531	0.006
Collins	1620	9064	0.007
DIP Scere	4584	20845	0.002
DIP Haspi	2523	3053	0.001

^*^The definition of graph density is given in [48].

Regarding the functional information of proteins, the GO terms in each functional category were obtained from the GO databases [20] for each of proteins. As there were some GO terms in the category of cellular components that may give any hints to what complex(es) a protein may belong to, we excluded them from our experiments.

For the ground truth of protein complexes in Saccharomyces cerevisiae, we used the curated protein complexes published by MIPS/CYGD [39] and CYC2008 [40] databases as of March 11, 2013. The numbers of curated protein complexes in MIPS/CYGD and CYC2008 were 255 and 408 respectively. After merging MIPS/CYGD and CYC2008, we obtained a total of 557 known protein complexes of Saccharomyces cerevisiae for performance evaluation. Concerning the human interaction network DIP Hsapi, the ground truth of protein complexes was obtained from the MIPS/CORUM [41] database, where we had 2835 known protein complexes of human beings.

### Preparations for performance evaluation

For the purpose of performance evaluation, we compared DCAFP with the state-of-the-art approaches including GMFTP [27], PCIA [19], MCL [10], MCODE [11], RNSC [12], CFinder [13], CMC [14], COACH [15] and IPCA [18]. Briefly speaking, for identifying protein complexes, GMFTP and PCIA considered the functional information of proteins and the graph topology of PPI network simultaneously while the other approaches used for comparison only made use of the graph topology of PPI network.

Regarding the parameter setting for each approach, we either adopted the default values provided by the corresponding software or performed many experimental trials to identify the values that obtained the best performance. The strategies of parameter setting for all approaches are listed in Table 2. In general, for any particular approach we considered, if the default settings of parameters were recommended by the authors in their original works, we adopted the strategy of default setting so that the default parameters as recommended were used in our experiments. Otherwise, we adopted the strategy of experimental trials to obtain, as much as possible, the parameter settings that would give the approach the best performance. To show how we performed experimental trials, we took DCAFP as an example. Given a PPI network, we chose the values of *w*_min_ and *os*_max_ varying from 0 to 1 with an interval 0.1 when tuning the performance of DCAFP and performed 10 trials for each combination of *w*_min_ and *os*_max_. We noted that the performance of DCAFP did not change much with proper values of *l*_max_ and *δ*. It is recommended to set *l*_max_ = 100 and *δ* = 1. In the experiments, matrices **A**_*p*_, **A**_*f*_, **A**_*c*_ and **D** were determined by Jaccard index.

**Table 2:** **The strategies of parameter setting for all approaches compared in experiments**

**Approach**	**Strategy**	**Approach**	**Strategy**
DCAFP	Experimental Trials	PCIA	Default Setting (Inflation = 1.8, μ = 0.7)
GMFTP	Default Setting (*k* = 1000)	MCL	Default Setting (Inflation = 1.8)
MCODE	Default Setting (VWP = 0.2)	RNSC	N/A
IPCA	Experimental Trials	CFinder	N/A
CMC	Experimental Trials	COACH	Experimental Trials

We used three independent evaluation measures, *f*-measure, Accuracy [9,27] and Maximum Matching Rate (MMR) [42], to compare the performances of all approaches. These three metrics are complementary to each other as they describe the performance from different perspectives.

For *f*-measure, following [11,19], a cluster identified is considered to be matched with a ground truth protein complex if the matching rate between them is not less than 0.2. The definitions of precision, recall and *f*-measure are given as:

13 $$ f-\mathrm{measure}=\frac{2\times \mathrm{Precision}\times \mathrm{Recall}}{\mathrm{Precision}\times \mathrm{Recall}}, $$

14 $$ \mathrm{Precision}=\frac{\mathrm{TP}}{\mathrm{TP}+\mathrm{F}\mathrm{N}}, $$

15 $$ \mathrm{Recall}=\frac{\mathrm{TP}}{\mathrm{TP}+\mathrm{F}\mathrm{P}}, $$

where TP (true positive) is the number of the identified complexes each of which has a ground truth complex matched, FP (false positive) is the number of the identified complexes each of which does not have a ground truth complex matched, and FN (false negative) is the number of ground truth complexes that are not matched by any of identified complexes.

Unlike *f*-measure where the minimum matching rate has to be specified in advance, the measures of Accuracy and MMR offer a natural and intuitive way to compare the predicted protein complexes with the ground truth protein complexes. In particular, Accuracy is defined as a geometric average of sensitivity [9] and positive predictive value [9] while MMR is to measure how accurately the predicted complexes represent the ground truth complexes.

Besides *f*-measure, Accuracy and MMR, we also adopted the function enrichment test to demonstrate the advantage of DCAFP with the introduction of functional preferences when compared with approaches such as PCIA that also made use of functional information to identify protein complexes. Given a threshold of p-value, an identified protein complex is functionally significant if at least one GO term shared among the proteins of this complex is found to be significantly enriched according to the functional enrichment test. In the experiments, for protein complexes identified in each of PPI networks, we used GO::TermFinder [43] to perform the function enrichment test with different thresholds of p-value.

### Comparison of *f*-measure, accuracy and MMR

Table 3 presents the overall performance of each approach in terms of *f*-measure, Accuracy and MMR when applied to identify protein complexes from PPI networks used in the experiments. As can be seen from Table 3, DCAFP consistently performed better among the best irrespective of the performance evaluation measures and PPI networks that were used in the experiment, and DCAFP is therefore a very promising approach for protein complex identification in PPI networks.

**Table 3:** **Results of** ***f*** **-measure, Accuracy and MMR**

**PPI network**	**Approach**	**#** ^**^**^	**Coverage** ^*^	***f*** **-measure**			**Accuracy**	**MMR**
				**Precision**	**Recall**	***f*** **-measure**		
Krogan	DCAFP	1195	1704	0.54	0.46	0.5^(1st)^	0.51^(2nd)^	0.23^(3rd)^
	PCIA	1210	2630	0.34	0.66	0.45^(2nd)^	0.5^(3rd)^	0.33^(1st)^
	GMFTP	297	1411	0.49	0.39	0.43	0.52^(1st)^	0.18
	MCL	545	2674	0.33	0.46	0.38	0.45	0.2
	MCODE	71	617	0.7	0.16	0.26	0.39	0.06
	RNSC	752	2145	0.33	0.57	0.42	0.46	0.27^(2nd)^
	IPCA	396	820	0.26	0.52	0.35	0.47	0.13
	CFinder	261	1140	0.73	0.31	0.44^(3rd)^	0.49	0.16
	CMC	297	939	0.44	0.34	0.38	0.49	0.15
	COACH	347	1056	0.59	0.33	0.42	0.48	0.17
Gavin	DCAFP	955	1176	0.56	0.38	0.45^(1st)^	0.46	0.19^(1st)^
	PCIA	268	1415	0.5	0.35	0.41	0.47^(3rd)^	0.14^(3rd)^
	GMFTP	161	917	0.69	0.32	0.44^(2nd)^	0.53^(1st)^	0.13
	MCL	189	1430	0.51	0.29	0.37	0.46	0.11
	MCODE	69	645	0.68	0.15	0.25	0.38	0.06
	RNSC	309	1262	0.47	0.42	0.44^(2nd)^	0.49^(2nd)^	0.17^(2nd)^
	IPCA	455	915	0.46	0.22	0.3	0.39	0.11
	CFinder	267	1124	0.71	0.3	0.42^(3rd)^	0.45	0.14^(3rd)^
	CMC	307	964	0.36	0.3	0.33	0.43	0.13
	COACH	322	1052	0.51	0.32	0.39	0.42	0.14^(3rd)^
Collins	DCAFP	1083	1271	0.69	0.44	0.54^(2nd)^	0.47	0.23^(3rd)^
	PCIA	494	1607	0.55	0.56	0.55^(1st)^	0.55^(3rd)^	0.27^(1st)^
	GMFTP	192	1160	0.67	0.37	0.48	0.57^(1st)^	0.16
	MCL	297	1620	0.61	0.5	0.55^(1st)^	0.56^(2nd)^	0.23^(3rd)^
	MCODE	111	857	0.82	0.28	0.42	0.53	0.12
	RNSC	356	1486	0.57	0.53	0.55^(1st)^	0.57^(1st)^	0.26^(2nd)^
	IPCA	312	938	0.51	0.22	0.31	0.35	0.12
	CFinder	318	1160	0.59	0.34	0.43	0.41	0.19
	CMC	174	1075	0.65	0.34	0.45^(3rd)^	0.52	0.15
	COACH	244	1114	0.57	0.34	0.43	0.4	0.16
DIP Scere	DCAFP	1643	2430	0.39	0.6	0.47^(2nd)^	0.46^(2nd)^	0.27^(3rd)^
	PCIA	1823	4440	0.26	0.72	0.38	0.44	0.004
	GMFTP	473	2407	0.39	0.49	0.43	0.47^(1st)^	0.2
	MCL	834	4579	0.23	0.45	0.3	0.36	0.19
	MCODE	62	795	0.44	0.09	0.15	0.27	0.03
	RNSC	1392	3791	0.22	0.67	0.33	0.39	0.28^(2nd)^
	IPCA	3682	4579	0.19	0.65	0.29	0.43	0.39^(1st)^
	CFinder	427	2143	0.58	0.43	0.49^(1st)^	0.45^(3rd)^	0.2
	CMC	1152	1775	0.29	0.56	0.38	0.45^(3rd)^	0.28^(2nd)^
	COACH	853	1952	0.39	0.52	0.45^(3rd)^	0.4	0.24
DIP Hsapi	DCAFP	1091	2124	0.39	0.29	0.333^(1st)^	0.32^(2nd)^	0.08^(2nd)^
	PCIA	855	2178	0.36	0.3	0.327^(2nd)^	0.33^(1st)^	0.001
	GMFTP	196	827	0.37	0.13	0.19	0.27	0.02
	MCL	556	2434	0.3	0.2	0.24	0.3	0.04
	MCODE	69	313	0.49	0.05	0.09	0.21	0.008
	RNSC	738	1846	0.33	0.25	0.28^(3rd)^	0.29	0.06^(3rd)^
	IPCA	1733	2434	0.19	0.23	0.21	0.33^(1st)^	0.11^(1st)^
	CFinder	134	515	0.64	0.13	0.22	0.31^(3rd)^	0.02
	CMC	136	417	0.58	0.13	0.21	0.28	0.02
	COACH	150	491	0.67	0.14	0.23	0.32^(2nd)^	0.02

^**^**^The total number of identified protein complexes.

^*^The total number of distinct proteins found in all identified complexes.

Regarding the number of protein complexes identified, MCODE tended to discover the fewest clusters for each of PPI networks and accordingly MCODE obtained a higher score of precision when compared with the other approaches. In contrast to MCODE, the number of protein complexes identified by DCAFP was subject to the density of PPI network. That is to say, when compared with the other approaches, DCAFP identified more protein complexes from dense PPI networks, such as Gavin and Collins, According to Table 4, we noted that the occurrences of overlapping clusters were more frequently observed in clusters identified by DCAFP in dense PPI networks, i.e., Gavin and Collins, than those identified in sparse PPI networks, i.e., DIP Scere and DIP Haspi, as the average percentage of pairs of overlapping clusters to all pairs of clusters identified in dense PPI networks was more than twice as much as that of sparse PPI networks. This observation was consistent with the conclusion made in [44], which pointed out that overlap becomes increasingly pervasive when networks are denser. Hence, the occurrences of overlapping complexes, to some extent, could account for the difference between dense and sparse PPI networks in the number of clusters identified by DCAFP.

**Table 4:** **Percentage of pairs of overlapping clusters to all pairs of clusters identified by DCAFP in each PPI network**

**PPI networks**	**Percentage of pairs of overlapping clusters**
Krogan	2.5%
Gavin	3.8%
Collins	4.6%
DIP Scere	2%
DIP Hsapi	1.1%

For DCAFP, although its scores of Precision and Recall were at the average level among all approaches according to Table 3, its performance on *f*-measure was better than the other approaches. In particular, DCAFP obtained the best *f*-measure scores for the PPI networks of Krogan, Gavin and DIP Hsapi and the second best *f*-measure scores for the remaining two PPI networks. When looking into the PPI networks of Collins and DIP Scere, we found that the difference between DCAFP and the approach with the best score of *f*-measure was much small, as DCAFP was only worse by 2% and 4% than the best approaches in Collins and DIP Scere respectively in terms of *f*-measure. Regarding Accuracy, DCAFP obtained a promising and stable performance in all PPI networks, as its score of Accuracy was always in the best three approaches with only a few exceptions. Similar results were also observed in the measure of MMR, where DCAFP also got competitive scores in each of PPI networks.

Concerning the effort of functional information to improve the performance of identifying protein complexes, we concentrated the discussion on DCAFP, PCIA and GMFTP, all of which additionally made use of functional information for clustering. From Table 3, we found that all these three approaches obtained a very competitive performance when applied to identify protein complexes. Although PCIA had a comparable performance in the PPI networks of Krogan, Gavin and Collins when compared with DCAFP, it performed worse than DCAFP in the remaining two PPI networks. When compared with GMFTP, DCAFP performed better in terms of *f*-measure and MMR with all PPI networks used in our experiments. When it came to Accuracy, GMFTP performed slightly better than DCAFP with all PPI networks expect with DIP Hsapi where DCAFP performed better. To understand why this was the case, we noted from the details of the clustering results that GMFTP tended to identify a small set of clusters for each of PPI networks. Hence, it is able for GMFTP to obtain a relatively higher positive predictive value especially when the size of protein complexes to be identified is also relatively small. Since this was indeed the case with the protein complexes in Saccharomyces cerevisiae, GMFTP was able to perform better in terms of Accuracy. However, in the case of protein complexes in Human, the Accuracy of GMFTP was not as good as the size of protein complexes in Human was much larger than that in Saccharomyces cerevisiae.

Overall, we noted that across all PPI networks, DCAFP yielded a promising performance. Comparing DCAFP with approaches that only considered the graph topology of PPI network, we found that DCAFP achieved a better performance than most of them for each of PPI networks as it additionally made use of functional information to improve the performance of identifying protein complexes. Moreover, DCAFP was better than both PCIA and GMFTP which also considered the functional information of proteins on average. Hence, if we intend to find an approach that can identify protein complexes more accurately, DCAFP is preferred.

### Impacts of density and functional preferences on the performance of DCAFP

In this section, we evaluated the impacts of the inclusion of information relating to density and functional preferences on the performance of DCAFP. To do so, we performed additional experiments with three versions of DCAFP and they were DCAFP with density only, DCAFP with functional preferences only and DCAFP with both. In particular, DCAFP with density only considered the density property while ignoring the property of functional preferences, DCAFP with functional preferences only considered the property of functional preferences while ignoring the density property, and DCAFP with both was the complete version of DCAFP. These three versions of DCAFP were tested with all of PPI networks and their results of *f*-measure, Accuracy and MMR were given in Table 5.

**Table 5:** **Performance comparison of three versions of DCAFP**

**PPI network**	**Version of DCAFP**	***f*** **-measure**			**Accuracy**	**MMR**
		**Precision**	**Recall**	***f*** **-measure**		
Krogan	Density only	0.77	0.29	0.42	0.49	0.16
	FPs only	0.62	0.22	0.32	0.41	0.1
	Both	0.54	0.46	0.5	0.51	0.23
Gavin	Density only	0.56	0.31	0.4	0.41	0.17
	FPs only	0.7	0.2	0.31	0.4	0.09
	Both	0.56	0.38	0.45	0.46	0.19
Collins	Density only	0.69	0.33	0.45	0.44	0.2
	FPs only	0.65	0.22	0.33	0.39	0.11
	Both	0.69	0.44	0.54	0.47	0.23
DIP Scere	Density only	0.52	0.31	0.39	0.39	0.17
	FPs only	0.27	0.62	0.38	0.39	0.2
	Both	0.39	0.6	0.47	0.46	0.27
DIP Hsapi	Density only	0.32	0.15	0.2	0.21	0.03
	FPs only	0.38	0.2	0.26	0.25	0.04
	Both	0.39	0.29	0.333	0.32	0.08

DCAFP with density only is the version of DCAFP that only considers the density property, DCAFP with functional preferences (FPs) only is the version of DCAFP that only considers the property of functional preferences, and DCAFP with both is the complete version of DCAFP.

Based on the results shown in Tables 4–5, DCAFP with density only performed better than most of the other approaches, but the performance of DCAFP with functional preferences only was not as well as that of DCAFP with density only in all PPI networks except DIP Hsapi. When compared with DCAFP with both, neither DCAFP with density only nor DCAFP with functional preferences only performed better than it. Hence, if only either density or functional preferences is considered, it is not sufficient for DCAFP to perform at its best and this is why both kinds of information are used when we formulate the optimization problem as given by (1).

When comparing the performance of DCAFP with density only with DCAFP with functional preferences only, we found that DCAFP with density only obtained a better performance in dense PPI networks, i.e., Gavin and Collins. However, the advantage of DCAFP with density only in sparse PPI networks was not as obvious as in dense PPI networks. In particular, the performance of DCAFP with density only was comparable to and worse than that of DCAFP with functional preferences only in DIP Scere and DIP Hsapi respectively. From this observation, we can say that the significances of density and functional preferences have to be considered differently when identifying protein complexes. However, for the current version of DCAFP, we do not take into consideration this point as it is yet to explore that which of them should be weighted more heavily than the other according to their significances given a PPI network. Since we believe that it is possible for us to improve the performance of DCAFP by considering the weight for each of term in (1), we would like to propose to investigate it as part of our future work.

### Comparison of functional enrichment with PCIA

In Table 6, we summarized the results of DCAFP and PCIA after performing functional enrichment tests with different thresholds of p-value in each of functional categories, values without brackets denoted the number of identified clusters that were functionally significant given a threshold of p-value while values within brackets denoted the percentage of functionally significant clusters to all identified clusters.

**Table 6:** **Results of functional enrichment test with different thresholds of p-value**

	**PPI network**	**Approach**	**< E-15**	**< E-10**	**< E-5**	**< E-2**
Λ_*p*_	Krogan	DCAFP	204 (17.1%)	346 (29%)	669 (56%)	796 (66.6%)
		PCIA	44 (3.6%)	97 (8%)	333 (27.5%)	561 (46.4%)
	Gavin	DCAFP	261 (27.4%)	409 (42.9%)	671 (70.4%)	766 (80.3%)
		PCIA	32 (11.9%)	64 (23.9%)	135 (50.4%)	165 (61.6%)
	Collins	DCAFP	506 (46.6%)	664 (61.2%)	902 (83.1%)	980 (90.3%)
		PCIA	59 (11.9%)	104 (21.1%)	284 (57.5%)	342 (69.2%)
	DIP Scere	DCAFP	167 (10.2%)	357 (21.7%)	870 (53%)	1093 (66.5%)
		PCIA	67 (3.7%)	140 (7.7%)	460 (25.2%)	761 (41.7%)
	DIP Hsapi	DCAFP	63 (5.8%)	169 (15.5%)	670 (61.4%)	806 (73.9%)
		PCIA	38 (4.4%)	104 (12.2%)	462 (54%)	590 (69%)
Λ_*f*_	Krogan	DCAFP	123 (10.3%)	213 (17.8%)	469 (39.2%)	656 (54.9%)
		PCIA	25 (2.1%)	60 (5%)	202 (16.7%)	378 (31.2%)
	Gavin	DCAFP	124 (13%)	225 (23.6%)	519 (54.5%)	673 (70.6%)
		PCIA	18 (6.7%)	39 (14.6%)	95 (35.4%)	129 (48.1%)
	Collins	DCAFP	303 (27.9%)	462 (42.6%)	746 (68.8%)	879 (81%)
		PCIA	32 (6.5%)	64 (13%)	186 (37.7%)	256 (51.8%)
	DIP Scere	DCAFP	85 (5.2%)	208 (12.7%)	571 (34.8%)	909 (55.3%)
		PCIA	30 (1.6%)	70 (3.8%)	265 (14.5%)	556 (30.5%)
	DIP Hsapi	DCAFP	32 (2.9%)	99 (9.1%)	481 (44.1%)	711 (65.2%)
		PCIA	13 (1.5%)	45 (5.3%)	269 (31.5%)	447 (52.3%)
Λ_*c*_	Krogan	DCAFP	279 (23.3%)	420 (35.1%)	701 (58.7%)	788 (65.9%)
		PCIA	60 (5%)	129 (10.7%)	307 (25.4%)	416 (34.4%)
	Gavin	DCAFP	320 (33.6%)	463 (48.6%)	708 (74.3%)	759 (79.6%)
		PCIA	41 (15.3%)	69 (25.7%)	129 (48.1%)	140 (52.2%)
	Collins	DCAFP	588 (54.2%)	703 (64.8%)	922 (85%)	970 (89.4%)
		PCIA	74 (15%)	123 (24.9%)	262 (53%)	305 (61.7%)
	DIP Scere	DCAFP	263 (16%)	466 (28.4%)	856 (52.1%)	1040 (63.3%)
		PCIA	87 (4.8%)	148 (8.1%)	384 (21.1%)	589 (32.3%)
	DIP Hsapi	DCAFP	44 (4%)	106 (9.7%)	421 (38.6%)	644 (59%)
		PCIA	27 (3.2%)	69 (8.1%)	268 (31.3%)	423 (49.5%)

Despite the differences between Human and Saccharomyces cerevisiae PPI network data, DCAFP identified much more complexes with functional significance than PCIA in all PPI networks as indicated by both number and percentage of protein complexes that passed the p-value tests. That is to say, even PCIA obtained a better performance of accuracy for some PPI network (i.e., Collins), the complexes identified by PCIA were less significant than those identified by DCAFP. The reason why DCAFP performed well in functional enrichment tests can be ascribed to the introduction of functional preferences, which is capable of emphasizing the functional homogeneity for each of attributes.

Also, the larger percentages of functional significant complexes identified by DCAFP can be an indicator that the complexes identified by DCAFP are real ones that could have been missed by laboratory-based identification techniques especially when DCAFP recalled the ground truth complexes well.

### Sensitivity tests of *w*_min_ and *os*_max_ on the performance of DCAFP

**Figure 2:**
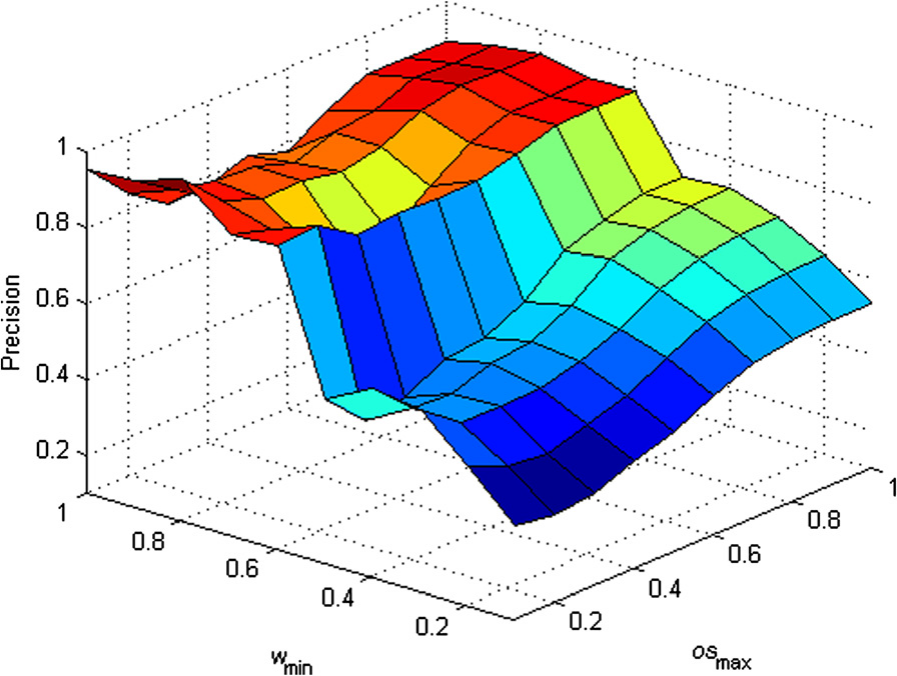
Sensitivity tests of *w*_min_ and *os*_max_ on the performance of DCAFP in terms of Precision.

**Figure 3:**
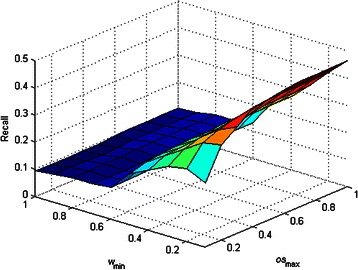
Sensitivity tests of *w*_min_ and *os*_max_ on the performance of DCAFP in terms of Recall.

**Figure 4:**
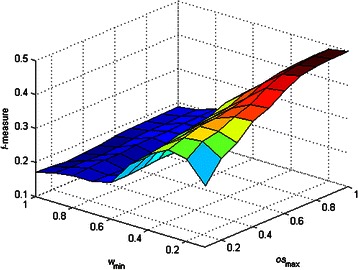
Sensitivity tests of *w*_min_ and *os*_max_ on the performance of DCAFP in terms of *f*-measure.

**Figure 5:**
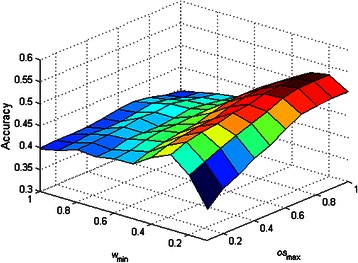
Sensitivity tests of *w*_min_ and *os*_max_ on the performance of DCAFP in terms of Accuracy.

**Figure 6:**
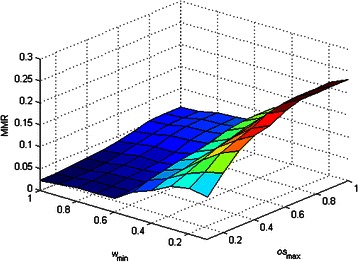
Sensitivity tests of *w*_min_ and *os*_max_ on the performance of DCAFP in terms of MMR.

As the performance of DCAFP is more concerned with the parameters *w*_min_ and *os*_max_, we concentrated on analysing the sensitivity tests of *w*_min_ and *os*_max_ on the performance of DCAFP in this section. During the experiments, we found that the effects of *w*_min_ and *os*_max_ were quite similar across all PPI networks, hence we took the PPI network of Krogan as an example to demonstrate how *w*_min_ and *os*_max_ affected the performance of DCAFP. Figures 2, 3, 4, 5 and 6 show the sensitivity tests of *w*_min_ and *os*_max_ on the performance of DCAFP in terms of Precision, Recall, *f*-measure, Accuracy and MMR respectively.

In Figure 2, we found that the increase in either *w*_min_ or *os*_max_ generally improved the precision of DCAFP, but such improvement was more sensitive to the change of *w*_min_ when compared with that of *os*_max_ according to the difference between the slope of the surface along *w*_min_ axis and that along *os*_max_ axis. When *w*_min_ is increasing, the condition of grouping a protein into a base cluster becomes more restricted and accordingly complexes identified by DCAFP become smaller in terms of the number of proteins. Similar to *w*_min_, an increasing *os*_max_ will make the condition of merging base clusters more restricted, thus resulting in smaller identified complexes. Since it is much easier for a small identified complex to have a ground truth complex matched, the precision of DCAFP can thus be improved.

In contrast to Figure 2, it is observed from Figure 3 that *w*_min_ play a more important role than *os*_max_ when affecting the performance of DCAFP in terms of Recall. In particular, the recall performance of DCAFP was improving with a falling *w*_min_, but changing the value of *os*_max_ did not have much effect on that. A possible reason for the weak effect of *os*_max_ is that the overlapping between base clusters was rarely found in Krogan.

Regarding the *f*-measure performance of DCAFP in Figure 4, the effects of *w*_min_ and *os*_max_ were similar to what we concluded from Figure 3. Based on Figures 3 and 4, we observed that the Recall score of DCAFP was more sensitive to the changes of *w*_min_ and *os*_max_ when compared with Precision. As *f*-measure considered Precision and Recall equally according to (13), its performance was more easily influenced by the one with high sensitivity. It was for this reason that *f*-measure and Recall scores of DCAFP reacted in a similar manner in Krogan. Hence, in the PPI network of Krogan, a better *f*-measure score of DCAFP was obtained with a small *w*_min_ and a large *os*_max_. Similar conclusions can also be made for Accuracy and MMR from Figure 5 and Figure 6 respectively.

In sum, the effect of *w*_min_ on the performance of DCAFP is more predictable, as *w*_min_ has an explicit impact to the size of complexes identified. But for *os*_max_, its effect on the performance of DCAFP is subject to the degree of overlapping found between base clusters. To put it more concretely, *os*_max_ will play a more important role in adjusting the performance of DCAFP if overlapping is more frequently observed in the base clusters; otherwise, it only has limited influence on the performance of DCAFP.

### Examples of overlapping protein complexes identified by DCAFP

**Figure 7:**
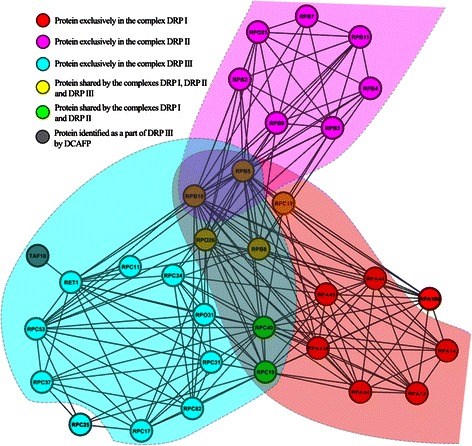
An example of network structure composed of three overlapping protein complexes DRP I, DRP II and DRP III in the PPI network of Krogan. Proteins are highlighted with different colors to indicate which complex(es) they belong to, and regions filled with different colors are the clusters identified by DCAFP. Protein symbols are used to name proteins.

**Figure 8:**
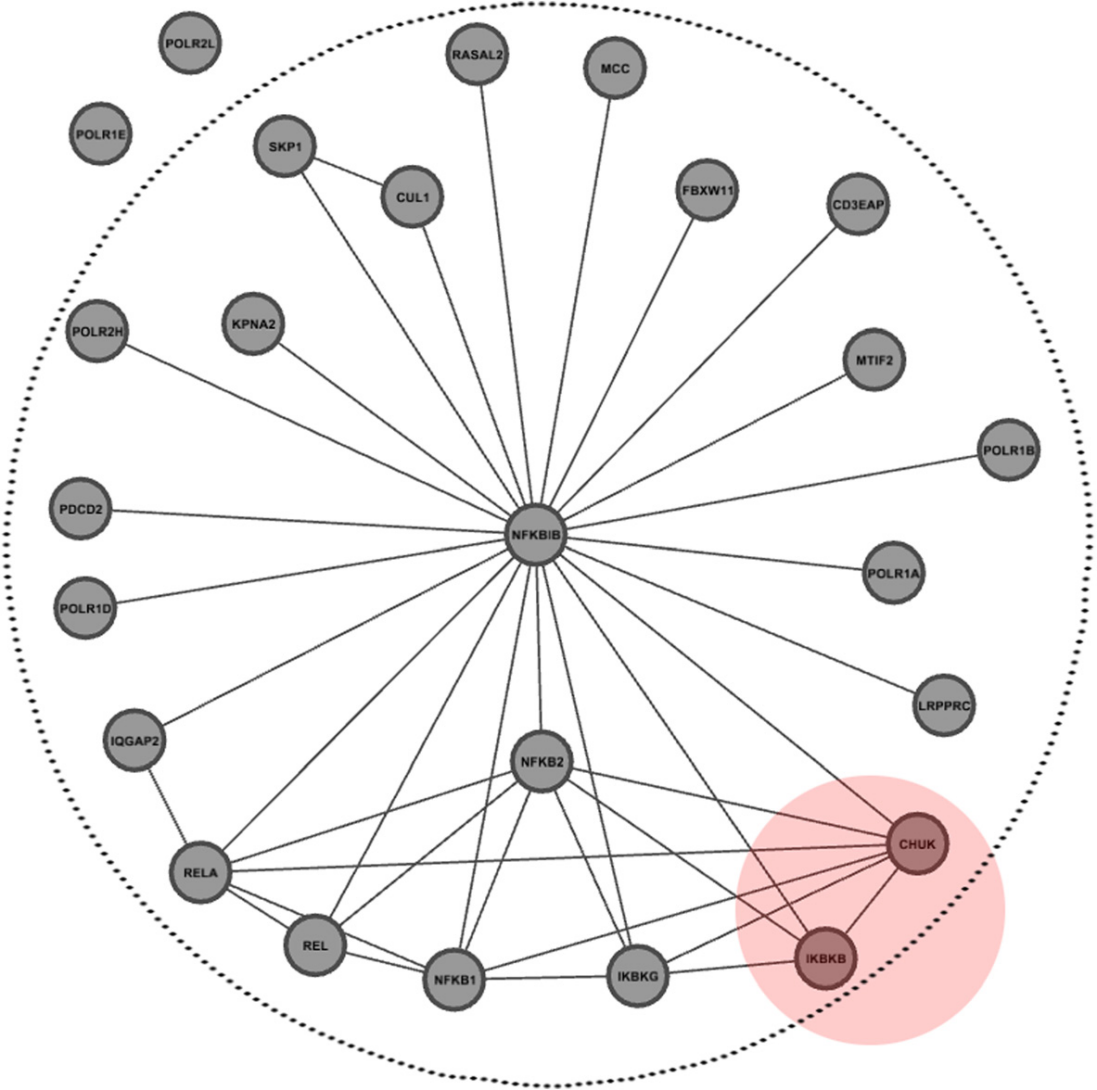
An example of network structure of the complex TNBSC in the PPI network of DIP Hsapi. Proteins in the dashed circle are those identified by DCAFP. Proteins in the region filled with red color constitute another protein complex, namely IKKA-IKKB complex, and all of them are identified by DCAFP. Protein symbols are used to name proteins.

To demonstrate the advantages of DCAFP when applied to predict protein complexes, we selected two examples of overlapping protein complexes identified by DCAFP from Krogan and DIP Hsapi respectively and illustrated them in Figure 7 and Figure 8 respectively. Besides, an in-depth analysis regarding the examples is also given below.

In the PPI network of Krogan, the network structures of three protein complexes, DNA-directed RNA polymerase I complex (DRP I), DNA-directed RNA polymerase II complex (DRP II), and DNA-directed RNA polymerase III complex (DRP III), were depicted in Figure 7. It can be observed from Figure 7 that all these three complexes had five proteins in common while DRP I and DRP II also shared two additional proteins. Hence, it is difficult for existing approaches to identify all of these three complexes because of their complicated structures. Regarding the performance of DCAFP in terms of identifying the three complexes in Figure 7, DCAFP successfully identified 9 out of 12 proteins, 13 out of 14 proteins and 15 out of 17 proteins from DRP I, DRP II and DRP III respectively as indicated by the regions filled with the colors of Red, Magenta and Cyan respectively. Hence, the high matching rates with DRP I, DRP II and DRP III can be an indicator of the promising performance of DCAFP.

Another point worth noting is about the protein TAF10 highlighted with grey colour in Figure 7. Although TAF10 was not verified to constitute DRP III in the CYC2008 database, DCAFP identified it as a part of DRP III ascribed to the reason that TAF10 and RET1, which was known as a part of DRP III according to the CYC2008 database, were similar with respect to the attributes of Λ_*p*_ and Λ_*c*_. Specifically speaking, when looking into the informative GO annotations that passed p-value test with a threshold of 0.01, we noted from the results that TAF10 and RET1 shared 27 out of 42, 1 out of 26 and 19 out of 35 annotations in the attributes of Λ_*p*_, Λ_*f*_ and Λ_*c*_ respectively. For TAF10 and RET1, the number of annotations shared in the attribute of either Λ_*p*_ or Λ_*c*_ was much more than that in Λ_*f*_.

Given a detailed literature review regarding the protein TAF10 [45,46], we noted that TAF10 was often involved in the transcription phase of RNA polymerase. Furthermore, the evidence from the update-to-date interaction database Interolog Finder [47] show that TAF10 was also interacting with proteins RPB8, RPB10, RPB5 and RPO26, all of which were verified to constitute DRP III according to CYC2008. Obviously, these interactions were not recorded in Krogan due to the experiment limitations at that time. Hence, we have reason to believe that TAF10 might have been missed in the laboratory experiments when DRP III was identified.

Another example of overlapping protein complexes is from the human PPI network DIP Hsapi as depicted in Figure 8. There were two protein complexes depicted in Figure 8, one was TNF-alpha/NF-kappa B signaling complex (TNBSC) and the other was IKKA-IKKB complex (IIC). Observing the topological structures of TNBSC and IIC, we found that both of them were not dense enough and IIC was completely overlapping with TNBSC. In this regard, even some approaches could identify either of them, few approaches were able to identify both of them. DCAFP addressed this problem with the use of **W**. In particular, based on the optimized result of **W**, the base cluster of TNBSC was originated from the protein NFKBIB while that of IIC started from the protein CHUK. Since the overlapping score between these two base clusters was too small to be merged in the last step of DCAFP, they were thus identified as the protein complexes. As a result, for TNBSC all proteins except POLR1E and POLR2L were identified by DCAFP, and IIC was completely identified by DCAFP. The reason why DCAFP could not identify POLR1E and POLR2L was that none of interactions involving these two proteins were found in the PPI network of DIP Hsapi.

## Conclusions

In this work, we have addressed the problem of identifying protein complexes by developing a new approach that considers the graph topology of PPI network and the functional information of proteins simultaneously. For the use of functional information, as we observed from the previous researches that proteins in a protein complex are rarely similar in all the categories of the functional information but instead they are normally found to be similar in specific subsets of the functional categories, functional preferences are thus introduced to emphasize such difference when identifying protein complexes. We then formulate the problem of identifying protein complex into a constrained optimization problem integrating the properties of functional preferences and dense structures of clusters. This constrained problem is then addressed by DCAFP in an iterative manner.

Experimental results on five PPI networks from the two species show the promising performance of DCAFP when applied to identify protein complexes. The comparison to the state-of-the-art approaches revealed that with the integration of functional preferences and dense structures, DCAFP exhibited improved performance with both in terms of accuracy of the identified complexes as well as in functional enrichment tests.

Regarding the future works, we would like to unfold it from two aspects. The first aspect is to consider assigning a weight to each of terms in the optimization problem of (1). As we found that the properties of density and functional preferences had different impacts to the performance of identifying protein complexes, the performance of DCAFP can be possibly improved if we make use of such difference by assigning different weights to the terms related to density and functional preferences. The other aspect is to implement DCAFP in a parallel manner so that the efficiency of DCAFP can be raised.

### Availability

The supporting datasets and the DCAFP software are available in http://www.comp.polyu.edu.hk/~cslhu/resources/dcafp.

### Endnote

^a^Density-based Clustering Approach with Functional Preferences.
